# Systemic proteomic and organ aging signatures associated with plasma Aβ oligomerization in a Korean cohort: a cross-sectional study

**DOI:** 10.3389/fnagi.2026.1620991

**Published:** 2026-03-23

**Authors:** Hyunjung Oh, Hongju Kim, Hojin Kang, Dohyeon Kwon, Leon French, Young Ho Park, Young Chul Youn, Seong Soo An, SangYun Kim, Sungmin Kang

**Affiliations:** 1 PeopleBio Inc., Seongnam-si, Gyeonggi-do, Republic of Korea; 2 Physiology Department and Donnelly Centre for Cellular and Biomolecular Research, University of Toronto, Toronto, ON, Canada; 3 Department of Neurology, Seoul National University Bundang Hospital and Seoul National University College of Medicine, Seongnam-si, Gyeonggi-do, Republic of Korea; 4 Department of Neurology, Chung-Ang University College of Medicine, Seoul, Republic of Korea; 5 Department of BionanoTechnology, Gachon University, Seongnam-si, Gyeonggi-do, Republic of Korea

**Keywords:** Alzheimer’s disease, blood proteome, dementia, oligomerization tendency, *β*-amyloid

## Abstract

**Background:**

Alzheimer’s disease (AD) is characterized by the accumulation of amyloid-beta (Aβ) in the brain, which begins decades before the appearance of clinical symptoms. Blood from AD patients, when spiked with synthetic Aβ, exhibited a higher Aβ oligomerization tendency (OAβT) than the non-AD subjects. OAβT reflected early pathological changes of AD and is considered as a promising blood-based biomarker. However, the mechanism underlying OAβT remained elusive. This study aimed to identify proteomic signatures associated with OAβT and explore its role in AD diagnosis.

**Methods:**

Forty AD and non-AD subjects from a Korean cohort were divided into four groups based on the disease diagnosis, OAβT values (thresholded at 0.78 ng/mL), and amyloid PET status (A-PET): A-PET-positive AD patients with high or low OAβT values, A-PET-negative non-AD subjects with high or low OAβT values. Using aptamer-based proteomics, 7,288 proteins from plasma samples were quantified, and the group differences were assessed in protein levels and the enrichment of gene sets associated with annotations from the Gene Ontology database. Further, we assessed whether OAβT-PET mismatched cases (A-PET-positive but OAβT-low or A-PET-negative but OAβT-high) exhibited distinct blood proteome signatures in comparison to typical AD cases. Aging signatures for 11 organs were analyzed to explore systemic factors linked to OAβT-PET discrepancies. Additionally, the pharmacological influences on the OAβT-related proteome were investigated by comparing OAβT-correlated proteins with a database of drug-induced proteomic changes.

**Results:**

Elevated OAβT values, regardless of AD diagnosis, correlated with increased immune response and decreased cellular metabolism. Dementia-predicting proteins were enriched in non-AD individuals with high OAβT. Accelerated muscle aging was associated with high OAβT values and worse cognitive function. Furthermore, several potential pharmacological modulators of OAβT, including Minocycline and Anamorelin, were identified.

**Conclusion:**

Our findings demonstrated OAβT as a reflection of systemic changes linked to early AD pathology. Moreover, the influence of medications and systemic aging on OAβT values pointed to the potential avenues for intervention and emphasized the importance of considering systemic factors in AD pathogenesis and treatment.

## 1 Introduction

Alzheimer’s disease (AD) is the most common type of dementia, characterized by the accumulation of amyloid plaques, neurofibrillary tangles, and brain atrophy. Amyloid-*β* (Aβ), the main component of amyloid plaques, is considered to play a crucial role in the pathogenesis of AD. Aβ self-aggregates into oligomers, protofibrils, and fibrils, initiating a cascade of events that leads to neuronal damage and cognitive decline. Among these, soluble oligomers and intermediate amyloids were regarded as the most toxic form, inducing neurodegeneration and cognitive deficit (Querfurth and LaFerla, 2010; Sakono and Zako, 2010; Benilova et al., 2012; Cline et al., 2018). Notably, Aβ deposition began more than 20 years before clinical symptoms appear (Villemagne et al., 2013). Early detection of these pathophysiological changes is crucial for effective intervention. Currently, amyloid positron emission tomography (PET) scans and cerebrospinal fluid (CSF) biomarker measurements are used to identify patients for monoclonal antibody therapies targeting amyloid pathology. However, their high costs, invasiveness, potential radiation exposure, and limited accessibility restrict widespread use. This calls for less invasive, cost-effective, and easily accessible biomarkers capable of detecting early-stage pathology.

While AD was traditionally viewed as a brain-specific disorder, a growing body of evidence suggested that AD involved systemic manifestations (Morris et al., 2014). The frequent co-occurrence of AD with conditions like diabetes mellitus, osteoporosis, and cardiovascular disease, established risk factors themselves, emphasized the interplay between systemic health and brain health (Arvanitakis et al., 2004; Whitmer et al., 2005; Wang et al., 2018). Given its accessibility and dynamic reflection of physiological changes, blood serves as an ideal medium for identifying and investigating these systemic alterations, supporting the importance of blood-based biomarkers in AD research.

Consistent with this perspective, human studies revealed substantial alterations in the blood proteome of AD patients (Voyle et al., 2015; Shi et al., 2019; Walker et al., 2021; Dammer et al., 2022; Jiang et al., 2022; Lindbohm et al., 2022). Similar to amyloid deposition, these systemic changes preceded dementia onset (Jia et al., 2024). Blood proteomic profiles were linked to 20-year dementia risk (Lindbohm et al., 2022) and to accelerated brain aging (Oh et al., 2023). A panel of 19 plasma proteins was also proposed for distinguishing dementia from non-demented individuals (Jiang et al., 2022).

Interestingly, blood from AD patients, when spiked with synthetic Aβ and incubated, exhibited higher levels of oligomeric Aβ compared to non-AD subjects (An et al., 2017). The blood Aβ oligomerization tendency (OAβT) demonstrated high accuracy in distinguishing AD from non-AD (An et al., 2017; Wang et al., 2017; Meng et al., 2019; Youn et al., 2020). It also strongly correlated with key AD biomarkers, including cortical atrophy, amyloid pathology, cognitive function, and CSF biomarkers, such as Aβ, phosphorylated-tau (Wang et al., 2017; Meng et al., 2019; Youn et al., 2019; Babapour Mofrad et al., 2021; Pyun et al., 2021; Kim, K.Y. et al., 2022; Wang et al., 2024). These findings demonstrate OAβT measurement as a promising tool for early diagnosis and disease monitoring. Its strong correlation with AD biomarkers suggests it may reflect underlying amyloid pathology in both central and peripheral systems.

Despite the strong association between high OAβT values and AD pathology, the molecular mechanisms driving this relationship remain unclear. Furthermore, while significant advances have been made in AD biomarker research, Asian populations remain underrepresented in large-scale proteomic studies. To address this gap, we focused on Korean individuals to provide population-specific insights into the molecular signatures of AD. Here, a plasma proteome analysis was conducted on Korean older adults with varying OAβT values to identify molecular signatures associated with OAβT. We first identified proteins and biological functions linked to a high Aβ oligomerization propensity and assessed whether AD-associated proteins are linked to increased OAβT values. Approximately 15% of subjects exhibited discrepancies between amyloid PET scan results and OAβT measurements (Pyun et al., 2021). Understanding these inconsistencies may reveal distinct disease subtypes or identify confounding systemic factors affecting amyloid aggregation in the periphery.

## 2 Materials and methods

### 2.1 Study participants

We used plasma samples from the Alzheimer’s Disease All Markers (ADAM) study cohort, which included subjects who visited Seoul National University Bundang Hospital between March 2019 and February 2021. PET imaging analyses were performed in these individuals to assess pathological features of AD, and PET images were visually evaluated as positive or negative by one nuclear medicine physician and two neurologists. The PET ligands used in this study included [^18^F]Florbetaben (*n* = 30), [^18^F]Flutemetamol (*n* = 6), [^11^C]Pittsburgh compound B (*n* = 1), and [^18^F]fluorodeoxyglucose (FDG, *n* = 3). All three subjects with FDG PET results were classified as PET negative. OAβT measurements, APOE genotyping, and neuropsychological tests including Mini-Mental State Examination (MMSE) and Clinical Dementia Rating (CDR) were also assessed.

**Figure 1:**
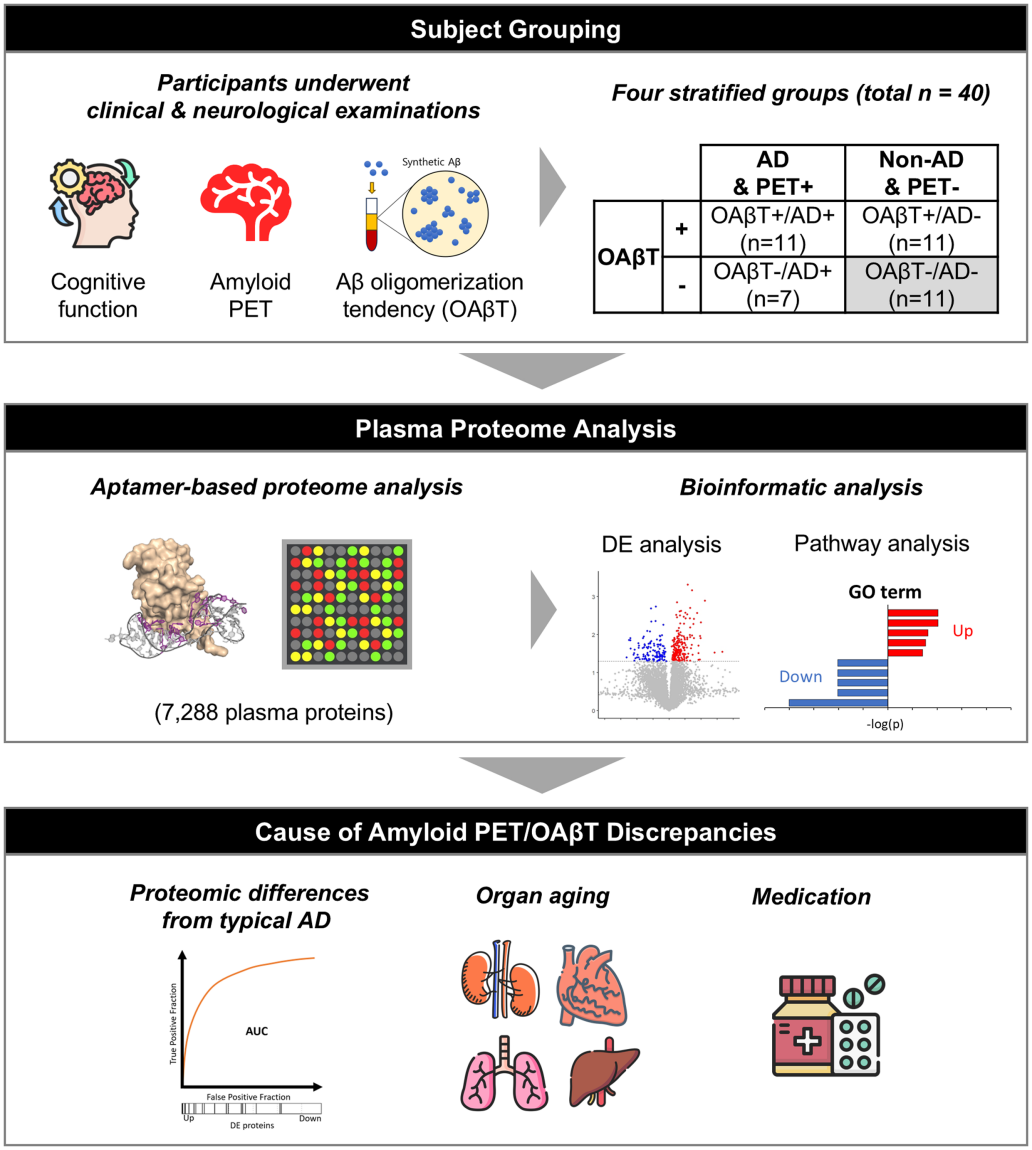
Study overview. 40 subjects were stratified based on their PET status, Aβ oligomerization propensity, and clinical diagnosis. 7,288 blood proteins were quantified utilizing aptamer-based proteomics and analyzed for differential expression, pathway enrichment, and compared with other proteomic datasets to assess AD signatures, organ aging, and the potential influence of drugs.

Forty subjects were selected based on clinical diagnosis, OAβT values, and PET status (Figure 1):

1) clinically AD patients with PET positivity and with high OAβT (OAβT+/AD+, *n* = 11)
2) clinically AD patients with PET positivity and with low OAβT (OAβT-/AD+, *n* = 7)
3) clinically non-AD patients with PET negativity and high OAβT (OAβT+/AD-, *n* = 11)
4) clinically non-AD subjects with PET negativity and low OAβT (OAβT-/AD-, *n* = 11)

Subjects were matched as closely as possible for age, sex, APOE genotype, and group sizes to minimize group differences.

The study protocol was reviewed and approved by the Institutional Review Board of the Seoul National University Bundang Hospital (IRB no. B-1811-507-006), and all subjects provided written informed consent.

### 2.2 Blood plasma preparation and measurement of Aβ oligomerization tendency in plasma

Blood samples were collected in sodium heparin tubes (Becton Dickinson vacutainer green cap), centrifuged at 1,500 g for 15 min at room temperature (RT). The resulting plasma was aliquoted and stored at −80 °C.

Aβ oligomerization tendency was measured using the AlzOn™ test (formerly MDS-OAβ or inBlood™ OAβ test) developed by PeopleBio Inc. This test employed the Multimer Detection System (MDS), a modified enzyme-linked immunosorbent assay (ELISA) that used epitope-overlapping antibodies to selectively detect oligomers over monomers. It primarily detected early-stage Aβ assemblies, particularly 7–35 mers (Choi et al., 2021).

Following the test protocol, plasma aliquots were thawed at RT for 15 min, mixed with PBR-1 (synthetic Aβ made by PeopleBio Inc.) and HAMA-blocker (Scantibodies Laboratory, Santee, CA, USA), and incubated at 37 °C for 48 h. The plasma samples and serially diluted standard Aβ oligomer samples were added to assay plates coated with 6E10 antibody (BioLegend, San Diego, CA, USA), which targeted amino acid residue 3–8 of human Aβ, and incubated at RT for one hour. After three washes, WO2-HRP antibody (Absolute Antibody Ltd., Oxford, UK), specific to amino acids 4–10 of human Aβ, was added and incubated for one hour at RT. Enhanced chemiluminescence substrate solution (Rockland Immunochemicals Inc., USA) was then added, and luminescence was measured. The concentrations of oligomerized Aβ were calculated using a standard curve with OAβT positivity defined as >0.78 ng/mL.

### 2.3 Assessment of plasma proteins

Plasma proteins were analyzed using the SomaScan version 4.1 assay (SomaLogic Inc., Boulder, CO, USA). Briefly, 7,288 plasma proteins were precipitated using single-stranded DNA aptamers that bound to specific protein targets and quantified via microarray containing DNA probes complementary to aptamer sequences (Rohloff et al., 2014). The specificity of the aptamers was confirmed using ELISA kits from Abcam (Cambridge, UK) for key protein hits: adiponectin (ab99968), TIMP-4 (ab113328), fibrinogen (ab108841), alpha-enolase (ab181417), IGFBP-2 (ab272207), and IGFBP-7 (ab213790).

### 2.4 Dataset processing

Normalization of the protein quantification levels from the SomaScan assay was performed by Somalogic Inc., using median signal normalization. Five calibrator samples were used to determine the scale factors. All samples were profiled on a single plate, eliminating the need for cross-run normalization. Samples flagged by the Somalogic Inc. preprocessing steps combined with principal component analysis (PCA) and Uniform Manifold Approximation and Projection (UMAP) plots were used to identify outlier samples (McInnes et al., 2018).

### 2.5 Statistical analysis

For the cross-sectional analysis, differences in protein levels between groups were tested with *t*-tests. To test for enrichment of gene sets of interest, the quantified proteins were sorted by their t-statistic values from the differential expression analysis. Within this ranking, the area under the receiver operating characteristic curve (AUC) was used to identify gene sets enriched in either direction (up-regulated: AUC > 0.5; down-regulated: AUC < 0.5). The Mann–Whitney U test was used to determine statistical significance with false discovery rate (FDR) correction for the number of gene sets tested. FDR-adjusted *p*-values were presented as q-values.

We tested enrichment of protein sets associated with AD, and Gene Ontology (GO) annotations. AD-associated proteins were sourced from two studies using the SomaScan platform (Walker et al., 2021; Lindbohm et al., 2022) and one study using the Olink Proteomics platform (Jiang et al., 2022). GO biological process and cellular component annotations were obtained from GO.db and org.Hs.eg.db packages (dated March 17, 2022) in R (v. 4.2.1). Only GO sets with 5–200 tested proteins were included, and overlapping GO groups were reduced using Rrvgo (similarity threshold = 0.9) (Sayols, 2023). Mutually overlapping gene sets were clustered together using EnrichmentMap (Merico et al., 2010), a Cytoscape plugin with an FDR *q*-value cutoff of 0.01, and edge similarity cutoff of 0.375.

Organ-specific biological age (organ age) was estimated using a plasma proteomics-based aging model^1^ previously developed by Oh et al. (2023). Briefly, organ-specific protein sets were defined based on transcriptomic data from the GTEx project. Genes exhibiting at least fourfold higher expression levels in a given organ in comparison with all other organs were classified as organ-enriched. These organ-enriched genes were mapped to SomaScan proteins to construct organ-specific input feature sets. For each organ, a LASSO regression model was trained to predict chronological age using plasma protein levels from cognitively unimpaired adults in the Knight Alzheimer’s Disease Research Center cohort. Protein expression values were *z*-score normalized, and sex was included as a covariate. Bootstrap aggregation was employed for model training with 500 LASSO models trained on resampled datasets. The regularization parameter was selected by five-fold cross-validation, favoring the most parsimonious model within 95% of the best performance. Individual organ age estimates were defined as the mean prediction across bootstrap models. Mediation analysis was performed with lavaan package (v. 0.6-19) in R.

To investigate whether the proteins most correlated with OAβT were possibly altered by specific drugs, we used a proteomic dataset profiling responses to 875 compounds (Mitchell et al., 2023). This study, conducted on the HCT116 human cancer cell line, measured protein expression 24 h after drug exposure. Each protein within the compound-induced proteome fingerprints was ranked from most upregulated to most downregulated (Supplementary Table 1 of Mitchell et al., 2023). The 100 most positively and negatively OAβT-correlated proteins were tested for enrichment within the rankings for each compound using the AUC metric. This analysis focused on proteins profiled by the SomaScan platform.

## 3 Results

### 3.1 Demographics and clinical characteristics

Of the initial 40 subjects, one outlier was identified and excluded from further analysis based on UMAP and PCA analyses of the proteome data. The remaining subjects had an average age of 68.8 ± 8.7 years with 21 females (53.8%) and 14 APOE ε4 carriers (35.9%). Baseline characteristics of the groups were summarized in Table 1. PET+ groups demonstrated lower MMSE scores and higher CDR and CDR-SOB scores. No significant differences were observed between the groups in age, sex, or education level.

**Table 1:** Demographics of the subjects used in the current analyses.

	OAβT+/AD+	OAβT-/AD+	OAβT+/AD-	OAβT-/AD-
Group size (number of females)	10 (6)	7 (4)	11 (5)	11 (6)
ApoE4 carrier	4 (40%)	2 (28.6%)	5 (45.4%)	3 (27.3%)
Education (years)	14.1 ± 4.2	15.7 ± 3.5	10.7 ± 3.8	12.5 ± 3.0
Age	69.7 ± 8.1	71.1 ± 7.7	70.6 ± 6.8	64.8 ± 10.9
OAβT (ng/mL)	1.1 ± 0.1	0.5 ± 0.2	1.0 ± 0.1	0.5 ± 0.2
MMSE	13.8 ± 4.5	14.6 ± 8.1	24.4 ± 4.1	24.6 ± 3.4
CDR (n of 0.5/1/2/3)	0/7/3/0	1/4/1/1	10/1/0/0	10/1/0/0
CDR-SOB	7.8 ± 2.4	7.9 ± 5.0	2.7 ± 2.1	2.7 ± 1.8

### 3.2 High OAβT-associated proteins and their biological function in matched cases

**Figure 2:**
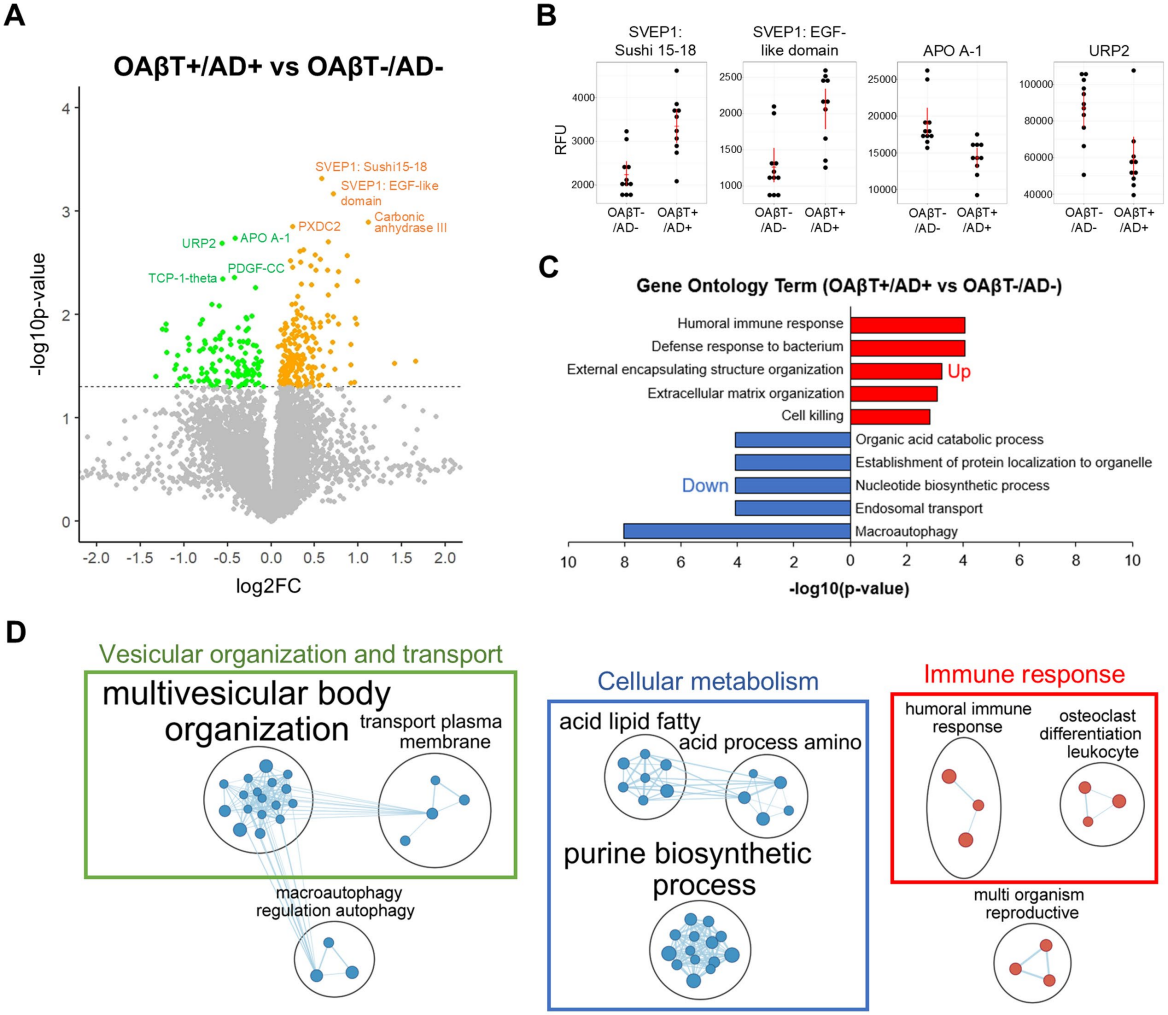
Changes of the plasma proteome in subjects with matched PET and OAβT results. **(A)** Volcano plot showing the differential protein expression of clinically AD patients with high OAβT value (OAβT+/AD+) vs. clinically non-AD patients with low OAβT value (OAβT-/AD-). **(B)** Dot plots of the 2 most upregulated (SVEP1: Sushi 15–18, SVEP1: EGF-like domain) and down-regulated proteins (APOA1, URP2) in OAβT+/AD+ compared to OAβT-/AD- group. Proteins were measured in relative fluorescence units (RFUs). Red lines represent the mean and standard error. **(C)** Representative up- or downregulated Gene Ontology (GO) terms of the OAβT-associated plasma proteins. Immune response-related biological pathways were significantly upregulated, cellular metabolism and autophagy-related biological pathways were downregulated in the blood plasma of AD patients with high OAβT values. **(D)** Cluster-based visualization of biological pathways altered in subjects with high OAβT values. The nodes represent gene sets corresponding to biological pathways with their sizes reflecting the number of genes they contain. The gene sets of the down- and up-regulated proteins are indicated in blue and red, respectively. Edges (lines connecting two nodes) depict the similarity between gene sets. Clusters identified through Markov clustering were labeled according to the semantic similarity of gene set names, using the AutoAnnotate package for Cytoscape. In AD subjects with high OAβT values, there was a notable downregulation in biological pathways associated with cellular metabolism, and vesicular organization, while immune response-related pathways were significantly upregulated.

Compared to OAβT-/AD- group, OAβT+/AD+ group displayed 321 differentially expressed (DE) proteins with 196 upregulated and 125 downregulated. Key upregulated proteins included SVEP1 (log2 fold change (LFC) = 0.58, *p* = 4.8×10^−4^), associated with AD pathogenesis (Walker et al., 2021), and TIMP-4 (LFC = 0.87, *p* = 2.7×10^−3^), which supported blood–brain barrier (BBB) integrity (Leco et al., 1997; Lopez-Navarro and Gutierrez, 2022). Proteins related to inflammation (e.g., α1-antitrypsin, macrophage mannose receptor, Kallikrein 14) (Oikonomopoulou et al., 2007; Emara et al., 2011; Martinez-Pomares, 2012; Ehlers, 2014) and oxidative stress (e.g., carbonic anhydrase III) (Cabiscol and Levine, 1995) were also identified. The top downregulated proteins included LRP (LFC = −0.82, *p* = 0.02), involved in Aβ clearance (Deane et al., 2008), along with several proteins related to protein folding, vesicular trafficking, apoptosis, and autophagy (Figures 2A,B, a full list of DE proteins is available in Supplementary Table S1).

GO analysis focused on biological processes revealed that the humoral immune response (*q* = 8.4×10^−5^) and defense response to bacterium (*q* = 8.4×10^−5^) were significantly upregulated in OAβT+/AD+ group in comparison to OAβT-/AD- group. In contrast, pathways related to macroautophagy (*q* = 9.5×10^−9^) and endosomal transport (*q* = 8.4×10^−5^) were significantly downregulated (Figure 2C). In total, 201 gene sets were significantly altered in OAβT+/AD+ subjects in comparison to OAβT-/AD- subjects with 69 upregulated, 132 downregulated gene sets (Supplementary Table S2). A pathway enrichment analysis summarized the output into three main themes: downregulated cellular metabolism (26 gene sets) and vesicular organization and transport (21 gene sets), and upregulated immune response-related pathways (6 gene sets; Figure 2D).

GO analysis focused on cellular components revealed an elevation of gene sets related to the postsynaptic membrane and downregulation of gene sets associated with microtubule, endosome, and autophagosome in OAβT+/AD+ group (Supplementary Table S3).

### 3.3 Blood proteome signatures of mismatched cases

Compared to OAβT-/AD- subjects, OAβT-/AD+ patients exhibited 427 DE proteins (202 upregulated, 225 downregulated), while OAβT+/AD- subjects displayed 311 DE proteins (232 upregulated, 79 downregulated; Supplementary Figure 1 and Table S1).

**Figure 3:**
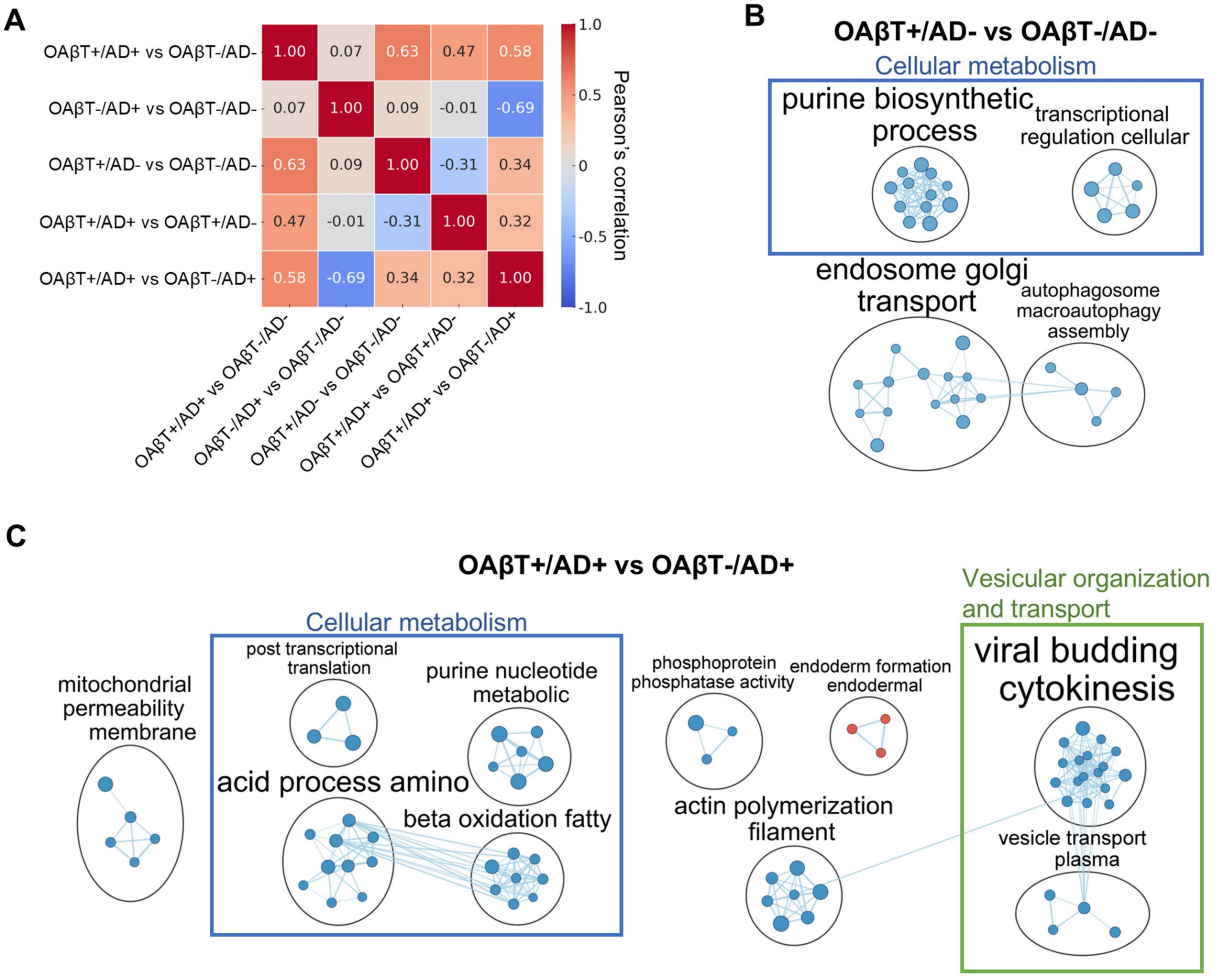
Alterations in the plasma proteome of subjects with mismatched PET and OAβT results**. (A)** The heatmap displays the correlation of effect sizes across different group comparisons. Similar gene alterations were observed when comparing high and low OAβT groups, regardless of disease status. **(B,C)** Gene set enrichment analysis. OAβT+/AD- group exhibited biological changes similar to those in OAβT+/AD+ group when compared to OAβT-/AD- group **(B)**. Likewise, comparable biological differences were observed between the OAβT+ and OAβT-/AD+ groups **(C)**.

Although the specific DE proteins varied across these comparisons (Supplementary Figure 2A; individual DE proteins in Supplementary Figures 3, 4), the overall DE profiles were similar when comparing high and low OAβT groups (Figure 3A). Pearson correlation analysis revealed a strong correlation in the proteome-wide *t*-statistics between OAβT+/AD+ vs. OAβT-/AD- and OAβT+/AD- vs. OAβT-/AD- (*r* = 0.63), as well as between OAβT+/AD+ vs. OAβT-/AD- and OAβT+/AD+ vs. OAβT-/AD+ (*r* = 0.58). In contrast, there was low correlation between OAβT+/AD+ vs. OAβT-/AD- and OAβT-/AD+ vs. OAβT-/AD- (*r* = 0.07), indicating distinct DE profiles.

OAβT+/AD- group exhibited biological changes similar to those seen in the OAβT+/AD+ when compared to the OAβT-/AD-, though with fewer enriched gene sets (Supplementary Figures 2B,C, Figure 3B). A total of 148 gene sets (35 upregulated, 113 downregulated) were significantly altered in OAβT+/AD- subjects in comparison to OAβT-/AD- subjects (Supplementary Table S2), with 17 gene sets associated with cellular metabolism being downregulated. Postsynaptic membrane-related gene sets were upregulated, while microtubule, endosome, and autophagosome-associated gene sets were downregulated in OAβT+/AD- subjects (Supplementary Table S3).

Similar biological differences between OAβT+ and OAβT-/AD+ groups were observed (Figure 3C). A total of 186 gene sets were significantly altered in OAβT+/AD+ subjects compared to OAβT-/AD+ subjects (61 upregulated, 125 downregulated; Supplementary Table S2), with significant downregulation in cellular metabolism-related (27 gene sets) and vesicular organization and transport-related (21 gene sets) pathways. Microtubule, endosome, and autophagosome-associated gene sets were downregulated in OAβT+/AD+ subjects compared to OAβT-/AD+ subjects (Supplementary Table S3). Despite the substantial number of DE proteins, no significant gene set in relation to biological function or cellular components was identified in the comparison between OAβT-/AD+ and OAβT-/AD- groups.

### 3.4 Possible causes of discordant PET and OAβT results

#### 3.4.1 Dementia-associated proteome

**Figure 4:**
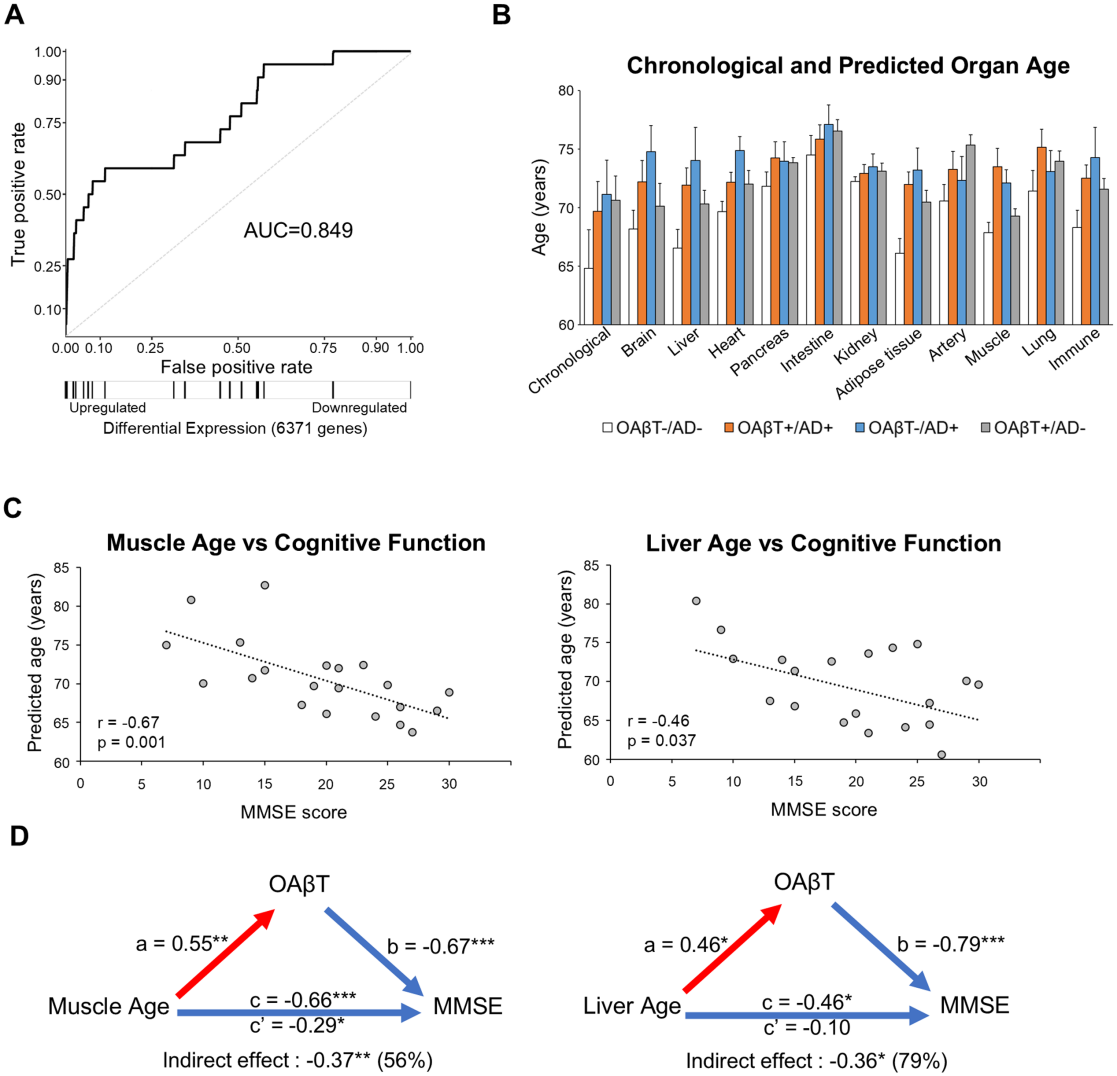
Investigation of potential factors contributing to the discordance between OAβT and PET results. **(A)** Enrichment of AD-associated proteins in non-AD subjects with high OAβT values. Receiver operating characteristic (ROC) curves illustrate the enrichment of 15 plasma proteins associated with an accelerated rate of cognitive decline and increased 20-year risk of dementia. Gene tags beneath the ROC curve indicate the location of these 15 dementia-associated plasma proteins within the ranked list of proteins, sorted by effect size. **(B)** Predicted organ ages in AD and non-AD subjects. Overall, AD subjects exhibited higher predicted ages compared to OAβT-/AD- subjects, regardless of OAβT values. **(C)** Correlations between organ age and MMSE scores in PET-OAβT matched cases. Among the examined organs, muscle and liver age were significantly correlated with MMSE scores. **(D)** Mediation analysis revealed that associations between muscle and liver age with cognitive function are strongly mediated by OAβT values. Red paths represent positive associations and blue paths represent negative associations. c = total effect, c’ = direct effect. *p < 0.05, **p < 0.01, ***p < 0.001.

First, we investigated whether mismatched cases exhibited distinct blood proteome profiles from those of typical AD patients by comparing our data with established blood proteome signatures associated with AD (Walker et al., 2021; Jiang et al., 2022; Lindbohm et al., 2022). We used a panel of 15 plasma proteins previously linked to accelerated cognitive decline and dementia risk to examine their association with elevated OAβT values (Lindbohm et al., 2022). In OAβT+/AD+ subjects, dementia-predicting proteins were significantly increased (AUC = 0.84, *q* = 8.8 × 10^−6^). These proteins were also elevated in OAβT+/AD- subjects (AUC = 0.85, *q* = 8.8 × 10^−6^; Figure 4A) and OAβT-/AD+ subjects (AUC = 0.79, *q* = 2.8 × 10^−4^).

BBB breakdown-related proteins from the panel were separately tested and significant increases were found in the OAβT+/AD+ (AUC = 0.86, *q* = 5.3 × 10^−4^), OAβT+/AD- (AUC = 0.85, *q* = 1.0 × 10^−3^), and OAβT-/AD+ (AUC = 0.83, *q* = 1.8×10^−3^) groups. Proteins associated with AD in other proteomic studies also showed significant changes across these groups (Supplementary Table S4).

To validate the proteomic findings, ELISA was used to assess six DE proteins. Significant increases in adiponectin, TIMP-4, and IGFBP-2 were confirmed in AD patients. While the expression levels of fibrinogen, alpha-enolase, and IGFBP-7 were significantly altered in subjects with high OAβT values as detected by SomaScan, ELISA results showed trend-level differences in the same directions (Supplementary Figure 5).

#### 3.4.2 Organ age

Given the recently reported association between accelerated organ aging and AD (Oh et al., 2023), we calculated the aging signatures for 11 organs to explore potential links to cases with mismatched OAβT and PET results (e.g., younger brain age in OAβT-/AD+ than OAβT+/AD+, or older brain age in OAβT+/AD- than OAβT-/AD-).

OAβT+/AD+ subjects exhibited significantly higher organ ages in adipose tissue (+2.3 years, *q* = 0.02) and muscle (+3.8 years, *q* = 0.03) in comparison to OAβT-/AD- subjects (Figure 4B). Similarly, OAβT-/AD+ subjects showed significantly higher organ ages in the heart (+3.8 years, q = 0.03), adipose tissue (+2.1 years, *q* = 0.03), and muscle (+1.0 year, *q* = 0.04) compared to OAβT-/AD- subjects. No significant difference was observed within the same diagnosis groups when separated by OAβT status (e.g., OAβT+/AD+ vs. OAβT-/AD+).

To identify organs contributing to cognitive function, we examined the correlations between predicted organ ages and neuropsychological test scores in participants with concordant PET and OAβT results, in order to minimize potential confounding effects arising from OAβT–PET discordance. Among the organs assessed, predicted ages of the muscle and liver showed significant correlations with MMSE scores as well as OAβT values (Figure 4C; full results in Supplementary Table S5). Notably, over half of the association between muscle and liver age and cognitive performance was mediated by OAβT (Figure 4D; full results in Supplementary Table S6).

#### 3.4.3 Medications

Aging is the primary risk factor for AD, and patients often experience age-related comorbidities (Wang et al., 2018). Both disease- and treatment-related changes are likely to influence the blood proteome in these individuals. Given that Aβ aggregation was influenced by various factors in the blood (Chaturvedi et al., 2015; Alam et al., 2016; Bode et al., 2018; Freyssin et al., 2018), we investigated the potential pharmacological influences on the OAβT-related proteome. By comparing drug-induced proteomic changes with the top 100 proteins positively and negatively correlated with OAβT values (Supplementary Table S7), we identified market-approved drugs that exhibited proteomic profiles similar to those associated with elevated OAβT (e.g., Minocycline, Ticagrelor), as well as drugs whose proteomic signatures were inversely correlated with OAβT levels, suggesting potential suppressive effects (e.g., Anamorelin; Table 2). The complete list of compounds was provided in Supplementary Table S8.

**Table 2:** Approved drugs that may regulate proteomic profiles associated with high OAβT.

Direction	Compound name	Primary target	AUC	*p-*value	Adjusted *p*-value	Usage
Mimicker	Minocycline	MMP9	0.652	3.55×10^−5^	0.031	Acne, respiratory infections, urinary tract infections, rheumatoid arthritis
	Ticagrelor	P2RY12	0.648	0.0000571	0.050	Acute coronary syndrome, prevention of thrombotic events
	Midostaurin	FLT3	0.634	0.000259	0.226	Acute myeloid leukemia, mastocytosis
	Benzbromarone	CYP2C9	0.631	0.000356	0.311	Gout
	Actonel (risedronate)	FDPS	0.625	0.000661	0.578	Osteoporosis, Paget’s disease
Reverser	Anamorelin	GHSR	0.349	0.0000389	0.034	Cancer cachexia (to increase appetite and body weight in cancer patients)
	Telotristat	TPH1	0.357	0.0000959	0.084	Carcinoid syndrome diarrhea in combination with somatostatin analog therapy
	Acitretin	RARG	0.358	0.000104	0.091	Psoriasis
	Rolapitant	TACR1	0.36	0.000138	0.121	Prevention of chemotherapy-induced nausea and vomiting
	Bosutinib	ABL1; SRC	0.368	0.000321	0.281	Chronic myeloid leukemia

## 4 Discussion

We found that Aβ oligomerization tendency was closely linked to the systemic changes associated with AD. The upregulation of immune response-related proteins in patients with high OAβT values indicated a significant role of inflammation in the pathophysiology of AD, consistent with previous studies identifying innate immune response-associated genes as risk factors (Kamboh et al., 2012). Furthermore, our findings of downregulation of cellular metabolism and autophagy pathways are consistent with reports linking impaired autophagy and metabolic dysregulation to toxic protein accumulation and subsequent cellular damage in AD (Nixon, 2013; Yan et al., 2020). An increase in postsynaptic membrane-associated components in patients with high OAβT values, regardless of cerebral AD pathology, suggests that BBB leakage is one of the earliest events in AD pathophysiology.

Plasma proteins concurrently changed in OAβT+/AD+ and OAβT+/AD- groups were of particular interest, as they may contribute to OAβT values. Upregulated proteins such as SVEP1, causally linked to AD (Walker et al., 2021), and fibrinogen, which directly bound Aβ, promoted Aβ fibrilization, and contributed to amyloid plaque accumulation and the BBB disruption, were particularly notable (Ahn et al., 2010). These findings suggest that OAβT values may reflect broader systemic factors contributing to AD pathophysiology, beyond what is captured by a single biomarker such as Aβ levels.

We also observed that subjects with high OAβT values exhibited signs of accelerated biological aging across multiple organs, particularly muscle. Predicted muscle age showed a strong association with both OAβT values and cognitive test performance. Carbonic anhydrase III, a muscle-enriched protein, was among the top three DE proteins in OAβT+/AD+ group. These findings were consistent with prior studies demonstrating associations between muscle mass, strength, and function with brain structure, Aβ burden, and cognitive function (Moon et al., 2018; Shaughnessy et al., 2020; Kim et al., 2024). Given the role of physical inactivity as a major risk factor of dementia (Hwangbo et al., 2023), and the beneficial effects of exercise on neuroplasticity through the upregulation of brain-derived neurotrophic factor (Erickson et al., 2012), systemic interventions targeting muscle health may reduce OAβT and slow AD progression.

In addition, we identified several drug compounds that may mimic or reverse OAβT-associated proteomic profiles. Interestingly, several cancer-related drugs emerged as potential reversers, aligning with our unpublished observation that AD patients with cancer tend to exhibit lower OAβT values. This could explain why OAβT-/AD+ subjects exhibit an AD-like proteomic signature without corresponding changes in biological pathways, as these drugs may alter specific OAβT-associated proteins rather than entire biological pathways. Further exploration of these medications may provide valuable insights into novel therapeutic strategies for AD.

Surprisingly, elevated OAβT values were associated with AD- and dementia-related proteome profiles, even in the absence of amyloid PET signal. This result suggested that systemic proteomic changes may precede cerebral amyloid deposition and earlier, upstream processes in AD pathogenesis. It was also noteworthy that MCI patients with higher OAβT values were more likely to progress to AD than MCI patients with low OAβT values (Xie et al., 2025). Since amyloid PET imaging primarily captured Aβ fibrils, which formed later than the most toxic Aβ oligomers, peripheral indicators, such as OAβT, may offer additional context regarding the disease state.

The biological basis for increased OAβT values in the blood of AD patients remains incompletely understood. Given that blood serves as a dynamic interface between peripheral organs and the brain, OAβT may be shaped by both central and peripheral factors that affect Aβ aggregation, clearance, or misfolding. Our findings, along with existing literature on proteomic changes and predicted organ age, supported the hypothesis that systemic aging, immune activation, and metabolic disturbances contribute to a peripheral environment conducive to Aβ oligomer formation.

While it remains unclear whether these systemic changes are causal or consequential, current evidence supports a bidirectional relationship. Human studies have revealed that blood proteomic alterations may precede the clinical onset of dementia (Lindbohm et al., 2022), as observed with cerebral amyloid deposition. Amyloid deposition during the preclinical stage may induce such systemic changes; conversely, systemic alterations may influence brain health by facilitating Aβ aggregation or impairing its clearance. Recent animal studies reinforce this perspective. In heterochronic parabiosis, where the circulatory systems of young and aged mice were surgically connected, young mice exposed to an aged systemic environment exhibited reduced neurogenesis (Villeda et al., 2011). Intravenous administration of whole blood or plasma from aged mice impaired hippocampal neurogenesis and cognitive function in young recipients (Villeda et al., 2011; Rebo et al., 2016; Yousef et al., 2019). In contrast, neutral blood exchange, in which 50 percent of plasma was removed from aged mice and replaced with saline with albumin, was shown to improve neurogenesis and cognitive function (Mehdipour et al., 2020; Mehdipour et al., 2021) with comparable benefits reported in human studies (Boada et al., 2017; Boada et al., 2022; Kim, D. et al., 2022). Whole-blood exchange using blood from young wild-type mice was also shown to reduce brain Aβ burden and improve memory in AD model mice (Urayama et al., 2022). Conversely, transferring blood from aged animals with extensive cerebral amyloid deposition to young AD model mice exacerbated amyloidosis and neuroinflammation (Morales et al., 2020). A similar trend toward increased brain amyloidosis was observed in young AD model mice receiving blood from aged WT mice. These findings collectively indicated the pivotal role of systemic factors in AD pathogenesis.

This study had several limitations. First, the small sample size and focus on an exclusively Asian population restricted the generalizability of our findings. Although our study provided valuable insights derived from a Korean cohort, including diverse populations in the future studies will be crucial to improve the generalizability and clinical applicability of emerging biomarkers, such as OAβT. Second, the pharmacological analysis relied on drug-induced proteomic profiles derived from HCT116 human cancer cells, which may not fully recapitulate the responses of neuronal or glial cells relevant to AD pathophysiology. Third, we also included three cases evaluated using FDG-PET rather than an amyloid PET tracer in the PET-negative groups. As FDG-PET was less sensitive than PIB-PET (Cohen and Klunk, 2014; Lesman-Segev et al., 2021), this may have contributed to inconsistencies between PET and OAβT. Finally, the cross-sectional design precluded causal inference. Longitudinal studies are needed to determine whether OAβT values can reliably predict future dementia development as well as Aβ deposition, and to evaluate how specific interventions, such as pharmacologic treatments or lifestyle modifications, affect blood proteome profiles and OAβT values. Future research should prioritize identifying the specific factors that accelerate Aβ oligomerization.

In conclusion, this study highlighted a strong link between Aβ oligomerization tendency and early systemic biological changes in AD, including augmented inflammation, reduced cellular metabolism, and impaired protein clearance. Furthermore, the influence of medications and lifestyle factors on OAβT values suggested potential therapeutic strategies to delay or prevent AD progression. These results emphasized the potential of OAβT measurements as a valuable biomarker for early AD diagnosis and personalized treatment.

## Data Availability

The datasets generated and analyzed in this study are publicly available in the FigShare repository and can be accessed at https://doi.org/10.6084/m9.figshare.31428272.

## Footnotes

1
https://pypi.org/project/organage/
