# Supplementary material for: Systemic proteomic and organ aging signatures associated with plasma Aβ oligomerization in a Korean cohort: a cross-sectional study
Source: Front Aging Neurosci. 2026 Mar 23;18:1620991. doi: 10.3389/fnagi.2026.1620991 (PMC13051358; doi:10.3389/fnagi.2026.1620991)
Supplement: Supplementary file 2 [file Presentation_1.PPTX]

## Slide 1
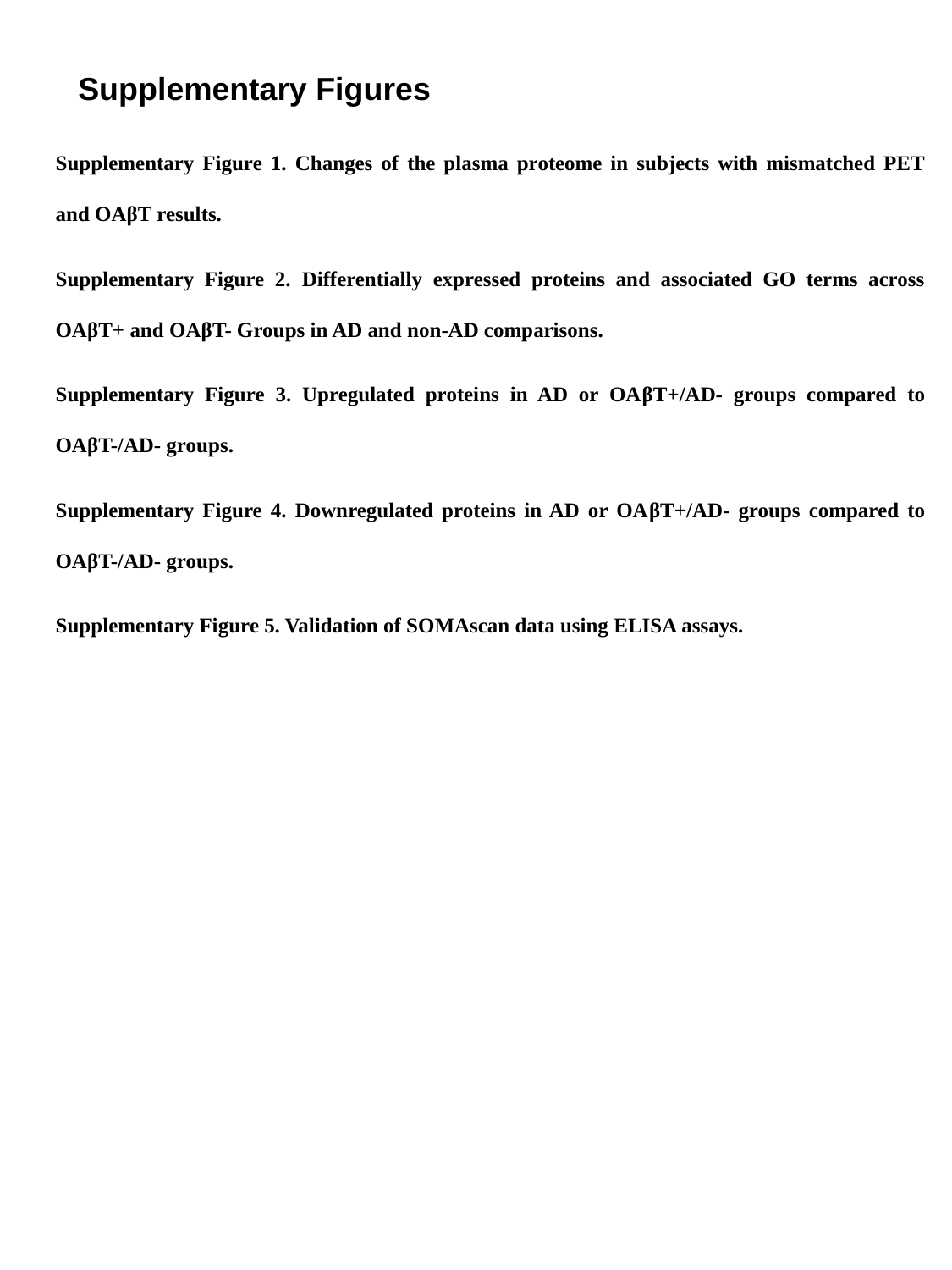

Supplementary Figures
Supplementary Figure 1. Changes of the plasma proteome in subjects with mismatched PET and OAβT results.
Supplementary Figure 2. Differentially expressed proteins and associated GO terms across OAβT+ and OAβT- Groups in AD and non-AD comparisons.
Supplementary Figure 3. Upregulated proteins in AD or OAβT+/AD- groups compared to OAβT-/AD- groups.
Supplementary Figure 4. Downregulated proteins in AD or OAβT+/AD- groups compared to OAβT-/AD- groups.
Supplementary Figure 5. Validation of SOMAscan data using ELISA assays.

## Slide 2
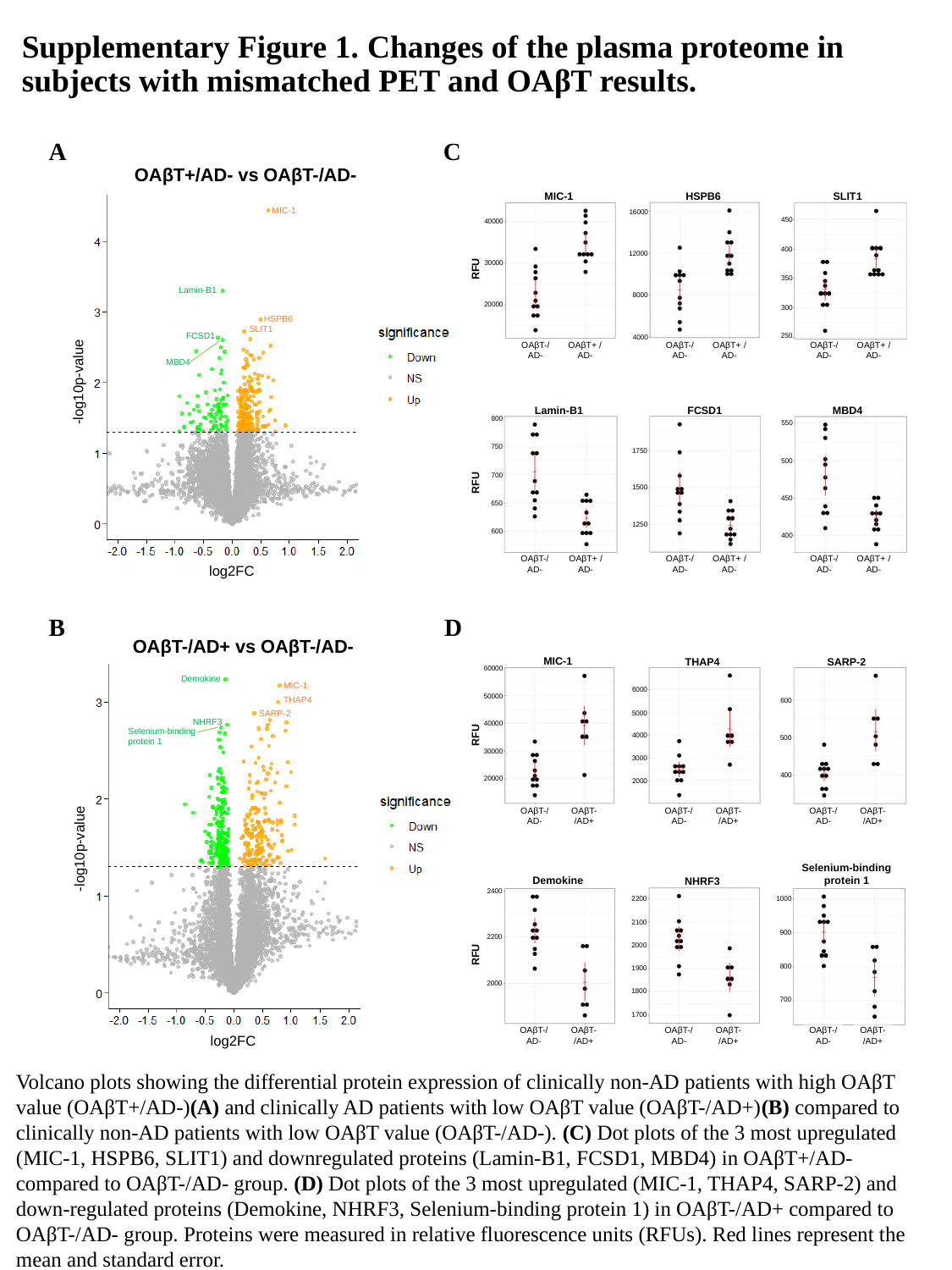

Supplementary Figure 1. Changes of the plasma proteome in subjects with mismatched PET and OAβT results.
A
C
OAβT+/AD- vs OAβT-/AD-
-log10p-value
log2FC
HSPB6
MIC-1
SLIT1
MIC-1
16000
450
40000
400
12000
RFU
30000
350
Lamin-B1
8000
20000
300
HSPB6
SLIT1
FCSD1
250
4000
OAβT-/AD-
OAβT+ /AD-
OAβT-/AD-
OAβT+ /AD-
OAβT-/AD-
OAβT+ /AD-
MBD4
MBD4
FCSD1
Lamin-B1
800
550
750
1750
500
RFU
700
1500
450
650
1250
600
400
OAβT-/AD-
OAβT+ /AD-
OAβT-/AD-
OAβT+ /AD-
OAβT-/AD-
OAβT+ /AD-
B
D
OAβT-/AD+ vs OAβT-/AD-
-log10p-value
log2FC
MIC-1
THAP4
SARP-2
60000
Demokine
MIC-1
6000
THAP4
50000
600
SARP-2
5000
NHRF3
Selenium-binding protein 1
40000
RFU
4000
500
30000
3000
400
20000
2000
OAβT-/AD-
OAβT- /AD+
OAβT-/AD-
OAβT- /AD+
OAβT-/AD-
OAβT- /AD+
Selenium-binding protein 1
Demokine
NHRF3
2400
2200
1000
2100
900
2200
2000
RFU
800
1900
2000
1800
700
1700
OAβT-/AD-
OAβT- /AD+
OAβT-/AD-
OAβT- /AD+
OAβT-/AD-
OAβT- /AD+
Volcano plots showing the differential protein expression of clinically non-AD patients with high OAβT value (OAβT+/AD-)(A) and clinically AD patients with low OAβT value (OAβT-/AD+)(B) compared to clinically non-AD patients with low OAβT value (OAβT-/AD-). (C) Dot plots of the 3 most upregulated (MIC-1, HSPB6, SLIT1) and downregulated proteins (Lamin-B1, FCSD1, MBD4) in OAβT+/AD- compared to OAβT-/AD- group. (D) Dot plots of the 3 most upregulated (MIC-1, THAP4, SARP-2) and down-regulated proteins (Demokine, NHRF3, Selenium-binding protein 1) in OAβT-/AD+ compared to OAβT-/AD- group. Proteins were measured in relative fluorescence units (RFUs). Red lines represent the mean and standard error.

## Slide 3
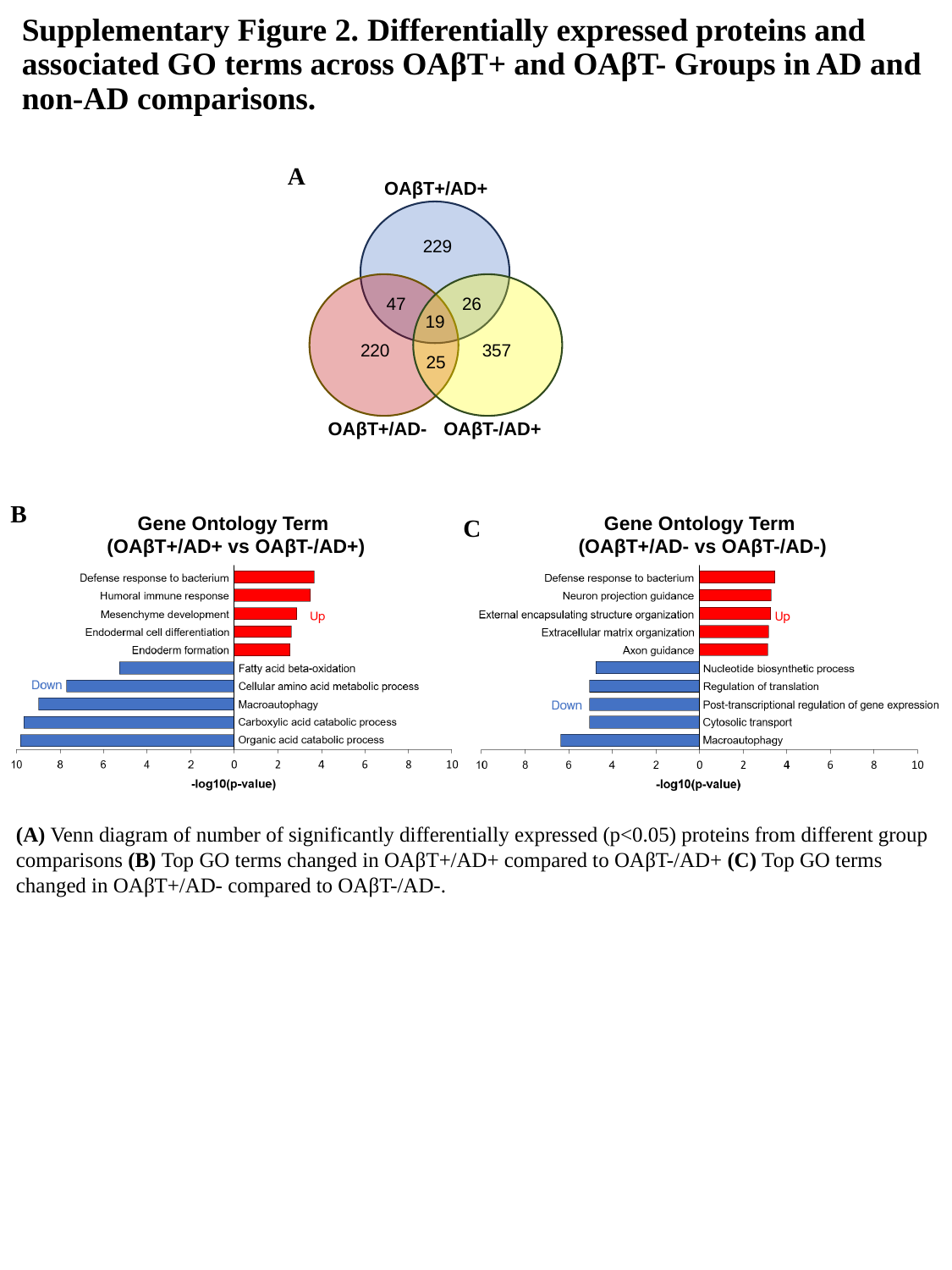

Supplementary Figure 2. Differentially expressed proteins and associated GO terms across OAβT+ and OAβT- Groups in AD and non-AD comparisons.
A
OAβT+/AD+
229
47
26
19
220
357
25
OAβT+/AD-
OAβT-/AD+
B
Gene Ontology Term
(OAβT+/AD+ vs OAβT-/AD+)
Gene Ontology Term
 (OAβT+/AD- vs OAβT-/AD-)
C
(A) Venn diagram of number of significantly differentially expressed (p<0.05) proteins from different group comparisons (B) Top GO terms changed in OAβT+/AD+ compared to OAβT-/AD+ (C) Top GO terms changed in OAβT+/AD- compared to OAβT-/AD-.

## Slide 4
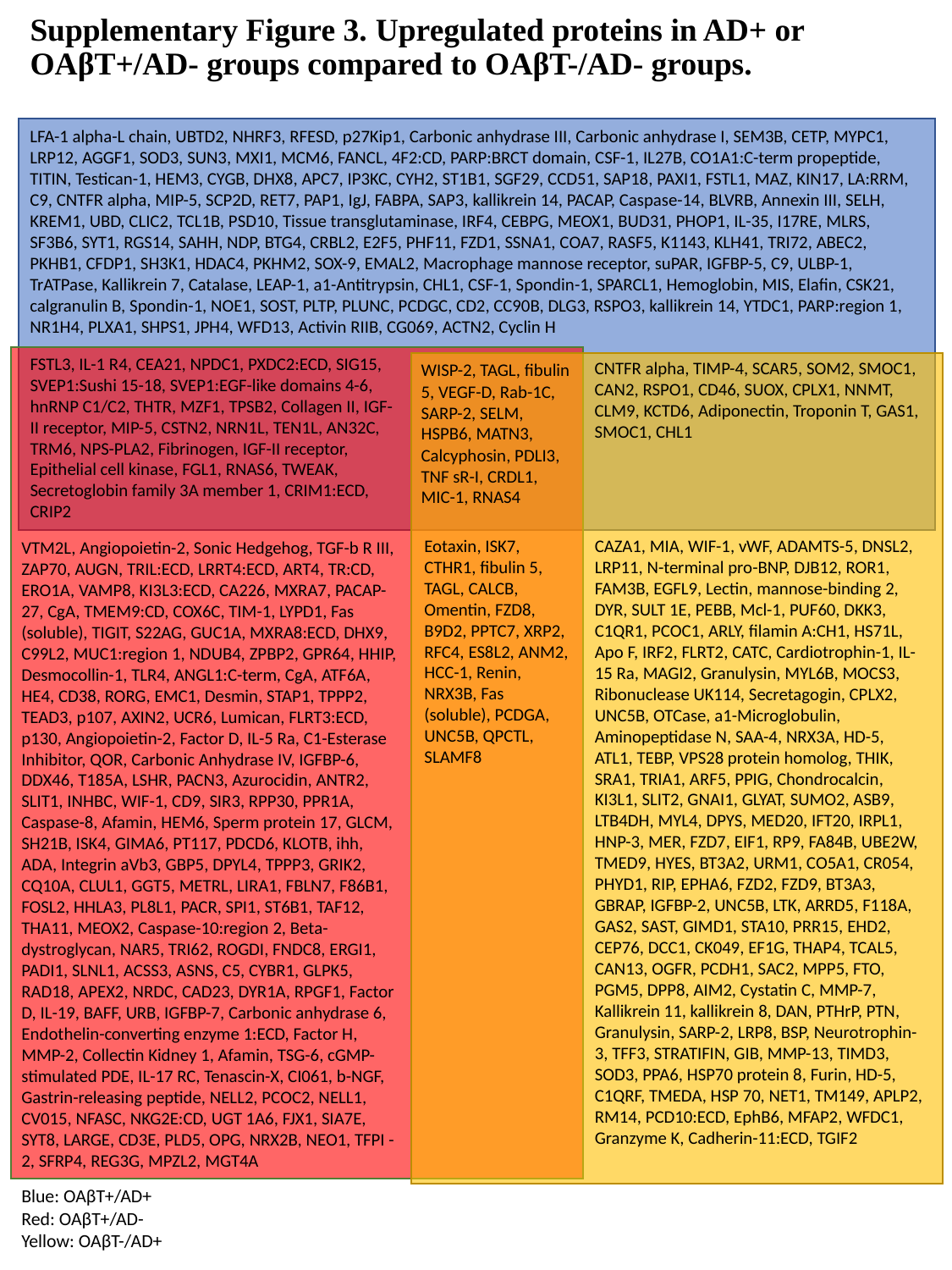

Supplementary Figure 3. Upregulated proteins in AD+ or OAβT+/AD- groups compared to OAβT-/AD- groups.
LFA-1 alpha-L chain, UBTD2, NHRF3, RFESD, p27Kip1, Carbonic anhydrase III, Carbonic anhydrase I, SEM3B, CETP, MYPC1, LRP12, AGGF1, SOD3, SUN3, MXI1, MCM6, FANCL, 4F2:CD, PARP:BRCT domain, CSF-1, IL27B, CO1A1:C-term propeptide, TITIN, Testican-1, HEM3, CYGB, DHX8, APC7, IP3KC, CYH2, ST1B1, SGF29, CCD51, SAP18, PAXI1, FSTL1, MAZ, KIN17, LA:RRM, C9, CNTFR alpha, MIP-5, SCP2D, RET7, PAP1, IgJ, FABPA, SAP3, kallikrein 14, PACAP, Caspase-14, BLVRB, Annexin III, SELH, KREM1, UBD, CLIC2, TCL1B, PSD10, Tissue transglutaminase, IRF4, CEBPG, MEOX1, BUD31, PHOP1, IL-35, I17RE, MLRS, SF3B6, SYT1, RGS14, SAHH, NDP, BTG4, CRBL2, E2F5, PHF11, FZD1, SSNA1, COA7, RASF5, K1143, KLH41, TRI72, ABEC2, PKHB1, CFDP1, SH3K1, HDAC4, PKHM2, SOX-9, EMAL2, Macrophage mannose receptor, suPAR, IGFBP-5, C9, ULBP-1, TrATPase, Kallikrein 7, Catalase, LEAP-1, a1-Antitrypsin, CHL1, CSF-1, Spondin-1, SPARCL1, Hemoglobin, MIS, Elafin, CSK21, calgranulin B, Spondin-1, NOE1, SOST, PLTP, PLUNC, PCDGC, CD2, CC90B, DLG3, RSPO3, kallikrein 14, YTDC1, PARP:region 1, NR1H4, PLXA1, SHPS1, JPH4, WFD13, Activin RIIB, CG069, ACTN2, Cyclin H
FSTL3, IL-1 R4, CEA21, NPDC1, PXDC2:ECD, SIG15, SVEP1:Sushi 15-18, SVEP1:EGF-like domains 4-6, hnRNP C1/C2, THTR, MZF1, TPSB2, Collagen II, IGF-II receptor, MIP-5, CSTN2, NRN1L, TEN1L, AN32C, TRM6, NPS-PLA2, Fibrinogen, IGF-II receptor, Epithelial cell kinase, FGL1, RNAS6, TWEAK, Secretoglobin family 3A member 1, CRIM1:ECD, CRIP2
CNTFR alpha, TIMP-4, SCAR5, SOM2, SMOC1, CAN2, RSPO1, CD46, SUOX, CPLX1, NNMT, CLM9, KCTD6, Adiponectin, Troponin T, GAS1, SMOC1, CHL1
WISP-2, TAGL, fibulin 5, VEGF-D, Rab-1C, SARP-2, SELM, HSPB6, MATN3, Calcyphosin, PDLI3, TNF sR-I, CRDL1, MIC-1, RNAS4
Eotaxin, ISK7, CTHR1, fibulin 5, TAGL, CALCB, Omentin, FZD8, B9D2, PPTC7, XRP2, RFC4, ES8L2, ANM2, HCC-1, Renin, NRX3B, Fas (soluble), PCDGA, UNC5B, QPCTL, SLAMF8
CAZA1, MIA, WIF-1, vWF, ADAMTS-5, DNSL2, LRP11, N-terminal pro-BNP, DJB12, ROR1, FAM3B, EGFL9, Lectin, mannose-binding 2, DYR, SULT 1E, PEBB, Mcl-1, PUF60, DKK3, C1QR1, PCOC1, ARLY, filamin A:CH1, HS71L, Apo F, IRF2, FLRT2, CATC, Cardiotrophin-1, IL-15 Ra, MAGI2, Granulysin, MYL6B, MOCS3, Ribonuclease UK114, Secretagogin, CPLX2, UNC5B, OTCase, a1-Microglobulin, Aminopeptidase N, SAA-4, NRX3A, HD-5, ATL1, TEBP, VPS28 protein homolog, THIK, SRA1, TRIA1, ARF5, PPIG, Chondrocalcin, KI3L1, SLIT2, GNAI1, GLYAT, SUMO2, ASB9, LTB4DH, MYL4, DPYS, MED20, IFT20, IRPL1, HNP-3, MER, FZD7, EIF1, RP9, FA84B, UBE2W, TMED9, HYES, BT3A2, URM1, CO5A1, CR054, PHYD1, RIP, EPHA6, FZD2, FZD9, BT3A3, GBRAP, IGFBP-2, UNC5B, LTK, ARRD5, F118A, GAS2, SAST, GIMD1, STA10, PRR15, EHD2, CEP76, DCC1, CK049, EF1G, THAP4, TCAL5, CAN13, OGFR, PCDH1, SAC2, MPP5, FTO, PGM5, DPP8, AIM2, Cystatin C, MMP-7, Kallikrein 11, kallikrein 8, DAN, PTHrP, PTN, Granulysin, SARP-2, LRP8, BSP, Neurotrophin-3, TFF3, STRATIFIN, GIB, MMP-13, TIMD3, SOD3, PPA6, HSP70 protein 8, Furin, HD-5, C1QRF, TMEDA, HSP 70, NET1, TM149, APLP2, RM14, PCD10:ECD, EphB6, MFAP2, WFDC1, Granzyme K, Cadherin-11:ECD, TGIF2
VTM2L, Angiopoietin-2, Sonic Hedgehog, TGF-b R III, ZAP70, AUGN, TRIL:ECD, LRRT4:ECD, ART4, TR:CD, ERO1A, VAMP8, KI3L3:ECD, CA226, MXRA7, PACAP-27, CgA, TMEM9:CD, COX6C, TIM-1, LYPD1, Fas (soluble), TIGIT, S22AG, GUC1A, MXRA8:ECD, DHX9, C99L2, MUC1:region 1, NDUB4, ZPBP2, GPR64, HHIP, Desmocollin-1, TLR4, ANGL1:C-term, CgA, ATF6A, HE4, CD38, RORG, EMC1, Desmin, STAP1, TPPP2, TEAD3, p107, AXIN2, UCR6, Lumican, FLRT3:ECD, p130, Angiopoietin-2, Factor D, IL-5 Ra, C1-Esterase Inhibitor, QOR, Carbonic Anhydrase IV, IGFBP-6, DDX46, T185A, LSHR, PACN3, Azurocidin, ANTR2, SLIT1, INHBC, WIF-1, CD9, SIR3, RPP30, PPR1A, Caspase-8, Afamin, HEM6, Sperm protein 17, GLCM, SH21B, ISK4, GIMA6, PT117, PDCD6, KLOTB, ihh, ADA, Integrin aVb3, GBP5, DPYL4, TPPP3, GRIK2, CQ10A, CLUL1, GGT5, METRL, LIRA1, FBLN7, F86B1, FOSL2, HHLA3, PL8L1, PACR, SPI1, ST6B1, TAF12, THA11, MEOX2, Caspase-10:region 2, Beta-dystroglycan, NAR5, TRI62, ROGDI, FNDC8, ERGI1, PADI1, SLNL1, ACSS3, ASNS, C5, CYBR1, GLPK5, RAD18, APEX2, NRDC, CAD23, DYR1A, RPGF1, Factor D, IL-19, BAFF, URB, IGFBP-7, Carbonic anhydrase 6, Endothelin-converting enzyme 1:ECD, Factor H, MMP-2, Collectin Kidney 1, Afamin, TSG-6, cGMP-stimulated PDE, IL-17 RC, Tenascin-X, CI061, b-NGF, Gastrin-releasing peptide, NELL2, PCOC2, NELL1, CV015, NFASC, NKG2E:CD, UGT 1A6, FJX1, SIA7E, SYT8, LARGE, CD3E, PLD5, OPG, NRX2B, NEO1, TFPI -2, SFRP4, REG3G, MPZL2, MGT4A
Blue: OAβT+/AD+
Red: OAβT+/AD-
Yellow: OAβT-/AD+

## Slide 5
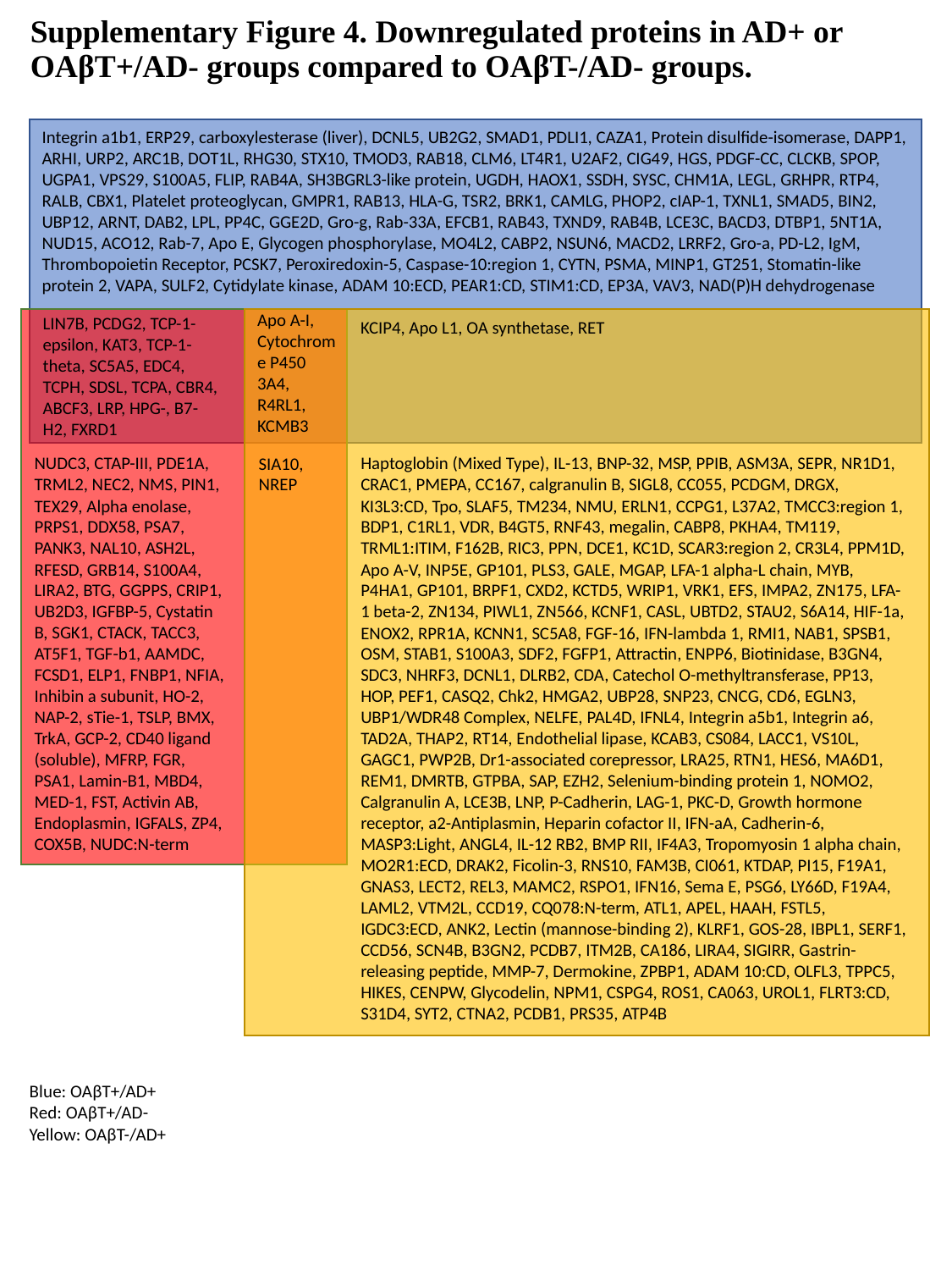

# Supplementary Figure 4. Downregulated proteins in AD+ or OAβT+/AD- groups compared to OAβT-/AD- groups.
Integrin a1b1, ERP29, carboxylesterase (liver), DCNL5, UB2G2, SMAD1, PDLI1, CAZA1, Protein disulfide-isomerase, DAPP1, ARHI, URP2, ARC1B, DOT1L, RHG30, STX10, TMOD3, RAB18, CLM6, LT4R1, U2AF2, CIG49, HGS, PDGF-CC, CLCKB, SPOP, UGPA1, VPS29, S100A5, FLIP, RAB4A, SH3BGRL3-like protein, UGDH, HAOX1, SSDH, SYSC, CHM1A, LEGL, GRHPR, RTP4, RALB, CBX1, Platelet proteoglycan, GMPR1, RAB13, HLA-G, TSR2, BRK1, CAMLG, PHOP2, cIAP-1, TXNL1, SMAD5, BIN2, UBP12, ARNT, DAB2, LPL, PP4C, GGE2D, Gro-g, Rab-33A, EFCB1, RAB43, TXND9, RAB4B, LCE3C, BACD3, DTBP1, 5NT1A, NUD15, ACO12, Rab-7, Apo E, Glycogen phosphorylase, MO4L2, CABP2, NSUN6, MACD2, LRRF2, Gro-a, PD-L2, IgM, Thrombopoietin Receptor, PCSK7, Peroxiredoxin-5, Caspase-10:region 1, CYTN, PSMA, MINP1, GT251, Stomatin-like protein 2, VAPA, SULF2, Cytidylate kinase, ADAM 10:ECD, PEAR1:CD, STIM1:CD, EP3A, VAV3, NAD(P)H dehydrogenase
Apo A-I, Cytochrome P450 3A4, R4RL1, KCMB3
LIN7B, PCDG2, TCP-1-epsilon, KAT3, TCP-1-theta, SC5A5, EDC4, TCPH, SDSL, TCPA, CBR4, ABCF3, LRP, HPG-, B7-H2, FXRD1
KCIP4, Apo L1, OA synthetase, RET
NUDC3, CTAP-III, PDE1A, TRML2, NEC2, NMS, PIN1, TEX29, Alpha enolase, PRPS1, DDX58, PSA7, PANK3, NAL10, ASH2L, RFESD, GRB14, S100A4, LIRA2, BTG, GGPPS, CRIP1, UB2D3, IGFBP-5, Cystatin B, SGK1, CTACK, TACC3, AT5F1, TGF-b1, AAMDC, FCSD1, ELP1, FNBP1, NFIA, Inhibin a subunit, HO-2, NAP-2, sTie-1, TSLP, BMX, TrkA, GCP-2, CD40 ligand (soluble), MFRP, FGR, PSA1, Lamin-B1, MBD4, MED-1, FST, Activin AB, Endoplasmin, IGFALS, ZP4, COX5B, NUDC:N-term
Haptoglobin (Mixed Type), IL-13, BNP-32, MSP, PPIB, ASM3A, SEPR, NR1D1, CRAC1, PMEPA, CC167, calgranulin B, SIGL8, CC055, PCDGM, DRGX, KI3L3:CD, Tpo, SLAF5, TM234, NMU, ERLN1, CCPG1, L37A2, TMCC3:region 1, BDP1, C1RL1, VDR, B4GT5, RNF43, megalin, CABP8, PKHA4, TM119, TRML1:ITIM, F162B, RIC3, PPN, DCE1, KC1D, SCAR3:region 2, CR3L4, PPM1D, Apo A-V, INP5E, GP101, PLS3, GALE, MGAP, LFA-1 alpha-L chain, MYB, P4HA1, GP101, BRPF1, CXD2, KCTD5, WRIP1, VRK1, EFS, IMPA2, ZN175, LFA-1 beta-2, ZN134, PIWL1, ZN566, KCNF1, CASL, UBTD2, STAU2, S6A14, HIF-1a, ENOX2, RPR1A, KCNN1, SC5A8, FGF-16, IFN-lambda 1, RMI1, NAB1, SPSB1, OSM, STAB1, S100A3, SDF2, FGFP1, Attractin, ENPP6, Biotinidase, B3GN4, SDC3, NHRF3, DCNL1, DLRB2, CDA, Catechol O-methyltransferase, PP13, HOP, PEF1, CASQ2, Chk2, HMGA2, UBP28, SNP23, CNCG, CD6, EGLN3, UBP1/WDR48 Complex, NELFE, PAL4D, IFNL4, Integrin a5b1, Integrin a6, TAD2A, THAP2, RT14, Endothelial lipase, KCAB3, CS084, LACC1, VS10L, GAGC1, PWP2B, Dr1-associated corepressor, LRA25, RTN1, HES6, MA6D1, REM1, DMRTB, GTPBA, SAP, EZH2, Selenium-binding protein 1, NOMO2, Calgranulin A, LCE3B, LNP, P-Cadherin, LAG-1, PKC-D, Growth hormone receptor, a2-Antiplasmin, Heparin cofactor II, IFN-aA, Cadherin-6, MASP3:Light, ANGL4, IL-12 RB2, BMP RII, IF4A3, Tropomyosin 1 alpha chain, MO2R1:ECD, DRAK2, Ficolin-3, RNS10, FAM3B, CI061, KTDAP, PI15, F19A1, GNAS3, LECT2, REL3, MAMC2, RSPO1, IFN16, Sema E, PSG6, LY66D, F19A4, LAML2, VTM2L, CCD19, CQ078:N-term, ATL1, APEL, HAAH, FSTL5, IGDC3:ECD, ANK2, Lectin (mannose-binding 2), KLRF1, GOS-28, IBPL1, SERF1, CCD56, SCN4B, B3GN2, PCDB7, ITM2B, CA186, LIRA4, SIGIRR, Gastrin-releasing peptide, MMP-7, Dermokine, ZPBP1, ADAM 10:CD, OLFL3, TPPC5, HIKES, CENPW, Glycodelin, NPM1, CSPG4, ROS1, CA063, UROL1, FLRT3:CD, S31D4, SYT2, CTNA2, PCDB1, PRS35, ATP4B
SIA10, NREP
Blue: OAβT+/AD+
Red: OAβT+/AD-
Yellow: OAβT-/AD+

## Slide 6
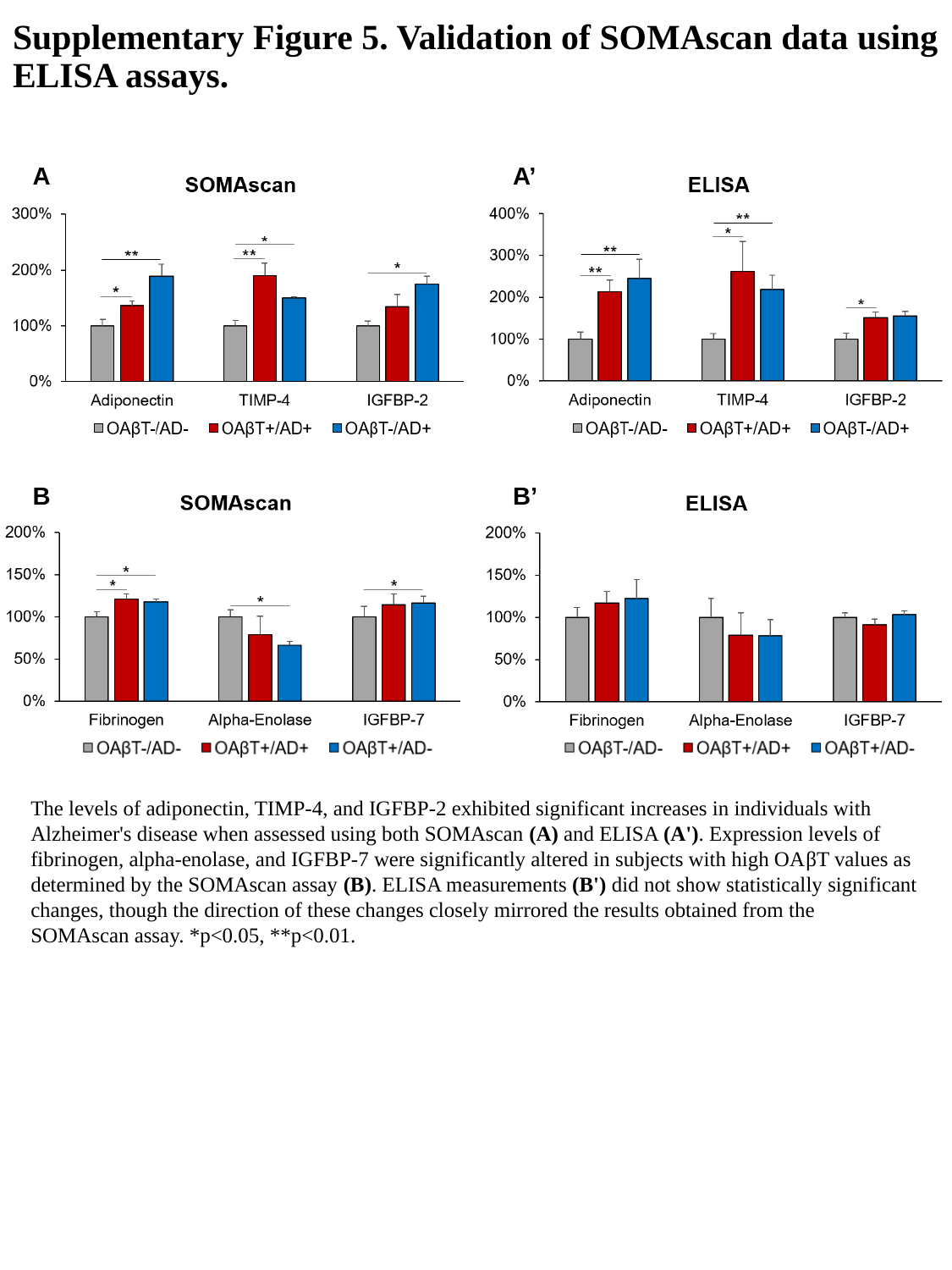

# Supplementary Figure 5. Validation of SOMAscan data using ELISA assays.
A
A’
B
B’
The levels of adiponectin, TIMP-4, and IGFBP-2 exhibited significant increases in individuals with Alzheimer's disease when assessed using both SOMAscan (A) and ELISA (A'). Expression levels of fibrinogen, alpha-enolase, and IGFBP-7 were significantly altered in subjects with high OAβT values as determined by the SOMAscan assay (B). ELISA measurements (B') did not show statistically significant changes, though the direction of these changes closely mirrored the results obtained from the SOMAscan assay. *p<0.05, **p<0.01.
